# Postsurgical oral symptoms after insertion of one or two implants for mandibular overdentures: short-term results of a randomized clinical trial

**DOI:** 10.1186/s40729-021-00321-2

**Published:** 2021-04-28

**Authors:** Gabriela Pereira de Resende, Ana Paula Dias, José Luiz Rodrigues Leles, João Antônio Chaves de Souza, Cláudio Rodrigues Leles

**Affiliations:** 1 Department of Oral Rehabilitation, School of Dentistry, Federal University of Goias, Praça Universitária, s/n, Setor Universitário, Goiania, Goias 74605-220 Brazil; 2 Private Practice, Goiania, Goias Brazil; 3 School of Dentistry, Campus Flamboyant, Paulista University, Rodovia BR 153, Km 503, s/n Fazenda, Marginal Botafogo, Goiania, Goias 74845-090 Brazil; 4 Department of Stomatology (Periodontology), School of Dentistry, Federal University of Goias, Praça Universitária, s/n, Setor Universitário, Goiania, Goias 74605-220 Brazil

**Keywords:** Mandibular overdenture, Randomized clinical trial, Postoperative pain, Implant-supported overdenture

## Abstract

**Objective:**

This randomized clinical trial aimed to compare the short-term postsurgical symptoms after insertion of one or two implants for retention of a mandibular overdenture. This study investigated whether the less invasive single-implant approach results in lower postoperative symptoms compared to the conventional two-implant overdenture.

**Materials and methods:**

Patients received new complete dentures and were randomly assigned to groups receiving one or two single-stage, early-loaded hydrophilic implants, inserted in the midline (*n* = 23), or the lateral incisor-canine area bilaterally (*n* = 24). Patient-reported postoperative symptoms were measured in a 0–100 visual analogue scale concerning pain in the surgical area, pain when chewing, bleeding, swelling, and unpleasantness. Data collection occurred 24 h and 7 and 21 days after surgery. Demographic and clinical features (smoking habit, classification of the residual ridges, and mucosal width and thickness at the implant sites), osteotomy for alveolar bone reduction, and surgery time were tested as predictors of symptom levels.

**Results:**

Overall reported symptoms were mild and self-limited, with high rates of complete remission after the early loading period of 3 weeks. Progressive improvement of symptoms occurred from the 24-h to the 7-day and 21-day follow-ups (*p* < 0.001), similarly in both groups. None of the clinical predictors was significantly associated with the changes in symptoms.

**Conclusions:**

Findings suggest that the insertion of one or two implants may result in similar postoperative outcomes.

**Clinical relevance:**

The severity of short-term postoperative symptoms may not be a critical factor for the decision between overdenture treatment with one or two implants.

## Introduction

There is a widely accepted consensus that a mandibular overdenture retained by two implants is the first implant treatment choice for edentulous subjects, resulting in better chewing function, comfort, and patient satisfaction when compared to the conventional complete denture [1]. However, previous reports suggest that the use of a single implant also yields successful outcomes comparable to the two-implant overdenture [2] and is considered a less costly intervention and demanding less invasive surgical procedures.

Since surgical anxiety is one of the main factors influencing patients’ refusal of dental implants [3, 4], reducing the number of implants to a minimum and the consequent reduction in costs and patient-perceived burdens related to implant surgery may represent positive aspects that increase patient adherence to treatment. Nevertheless, the hypothesis that the use of a single implant results in a clinically relevant reduction in postsurgical symptoms has not been tested before. Moreover, postoperative pain or discomfort may affect daily activities such as oral hygiene, eating or conversation, and difficulty using a removable prosthesis during the surgical healing period. Therefore, there is a need to assess the impacts of the implant insertion surgery and the associated bone and soft tissue injuries that lead to postoperative inflammation, edema, and pain in the short term [5].

Patient report of postoperative pain after surgical placement of dental implants is considered to be mild to moderate [6, 7] with peaks after 6 h [8], and may withstand for up to 3 days after surgery [9]. Postoperative symptoms can be controlled using analgesic and anti-inflammatory drugs, proper oral hygiene, physical therapy, rest, and minimizing trauma to the tissue healing area [10]. Postoperative pain is also markedly influenced by the surgical technique and is less intense with minimally invasive or flapless approaches [8, 11, 12].

Considering that a single-implant overdenture is assumed to be less extensive and invasive than a 2-implant overdenture, we hypothesized that it could be associated with reduced tissue manipulation, shorter surgical time, and lower incidence and severity of postoperative bleeding, pain, and discomfort. Hence, this study aimed to compare the short-term patient-reported postsurgical outcomes after implant surgery for treatment with mandibular overdenture retained by one or two implants. The underlying study question is whether 1-IOD surgery results in lower postsurgical symptoms compared to the 2-IOD.

## Methods

### Study design

This study reports the short-term postsurgical symptoms as part of a randomized clinical trial comparing the treatment with mandibular overdentures retained by a single (1-IOD group) or two implants (2-IOD group) opposing to a conventional maxillary complete denture. The study protocol was previously registered (NCT03691285) and approved by the local ethical research committee (CAEE 65240617.5.0000.5083). A detailed description of the randomized clinical trial is previously published [13], which includes the 1-year results of comparative changes in oral health-related quality of life, patient satisfaction, and chewing function. All patient treatments and data collection were conducted at the School of Dentistry of the Federal University of Goias, Brazil.

### Participants

Participants were fully edentulous subjects needing replacement of old complete dentures and implant intervention for retention of the mandibular denture. Eligibility criteria included favorable general health and sufficient bone dimensions for placement of an implant at least 8-mm length in the potential implant sites (midline and canine regions), assessed using a panoramic radiograph. The exclusion criteria comprised any general or local contra-indication for implant treatment, presence of oral conditions that demand additional treatments, subjects with significant cognitive decline, and those who disagreed to be randomly assigned to one of the two study groups. Subjects who did not meet the inclusion and exclusion criteria were referred for appropriate treatment in other clinical settings of the dental school. The study sample size was calculated within the context of the main study [13] and comprised 48 participants, 24 in each group.

After complete denture treatment, participants were randomly assigned to the study groups (1-IOD and 2-IOD groups) using block randomization and stratification by gender, aiming to reduce allocation bias and to achieve a balance of participants in the two groups. To avoid selection bias, each participant’s assignment group was labeled and concealed in sealed black envelopes, and the group identification was only revealed at the time of the implant surgery.

### Intervention

A new set of complete dentures was provided to all participants. They were scheduled for implant treatment planning after reporting being adapted to denture use with no major complaints, except the perceived need to improve the mandibular denture retention and stability.

Implant surgery planning included the clinical assessment of the residual alveolar dimensions in the midline and bilateral canine areas and panoramic radiograph for confirmation of sufficient bone height for placement of one or two regular diameter implants with 8-, 10-, or 12-mm length. Preoperative care included verification of vital signs, medication with antibiotic prophylaxis with amoxicillin 2 g or clindamycin 600 mg, paracetamol 750 mg, and dexamethasone 8 mg in cases of alveolar ridge reduction [14].

At the time of the surgery, the group assignment for each participant was disclosed to the patient and the surgeon. Implant surgery procedures initiated infiltration anesthesia with articaine hydrochloride with 1:100,000 epinephrine, crestal incision allowing a full-thickness flap elevation to expose the implant site extending between canine regions for the 1-IOD group and immediately beyond the mental foramen bilaterally for the 2-IOD group. When needed, alveolar bone reduction was performed for preparation of the implant site with a straight surgical handpiece and a tungsten drill under sterile saline irrigation. In all cases, a surgical guide was obtained by duplication of the mandibular denture, which served as a reference for correct implant positioning.

Tissue level Straumann® Standard Plus SLActive® regular neck implants (Straumann 033.561S/652S/563S, Institute Straumann AG, Basel, Switzerland) were inserted in the mandible midline (1-IOD group) or the lateral incisor-canine area bilaterally (2-IOD group). Drilling sequence and implant insertion were performed according to the manufacturer’s protocol. The final insertion torque was checked with a torque wrench adjusted to 25 N cm. After implant placement and healing abutment connected to the implants, tissues were sutured, and the mandibular denture was relieved and relined with temporary soft relining material (Soft Comfort, Dencril, São Paulo, Brazil). The patient’s postoperative care included paracetamol 750 mg in case of pain (every 6 h for up to 3 days), a soft diet, and 0.12% chlorhexidine mouthwash rinse for 1 week. Sutures were removed after 7 days.

### Outcomes

#### Postoperative pain and discomfort

After the implant surgery, postoperative pain and discomfort were measured by questionnaires using graduated visual analogue scales (VAS) from 0 to 100 mm. Each participant was asked to rate the value that corresponds to their perception of the following aspects: (1) pain in the surgical area, (2) pain when chewing, (3) bleeding in the surgical area, (4) swelling of the surgical area, and (5) perception of the unpleasantness of the surgery [15]. Data were collected at 24 h and 7 and 21 days after implant placement surgery.

### Independent variables

Data concerning participants’ demographic (age and sex) and clinical features were collected. Clinical characteristics included smoking habit, classification of the residual ridges (high well-rounded, knife-edge, flat, depressed) [16], and prognostic classification scores (Prosthodontic Diagnostic Criteria) according to the American College of Prosthodontists (ACP) for edentulous patients [17].

The mucosal width and thickness at the implant sites were evaluated before the surgical procedures to record the keratinized tissue width (KTW) and vertical mucosa thickness (VMT). The KTW was measured by identifying the mucogingival junction at the buccal, lingual, and crestal sites using a calibrated probe and the rolling technique. Measurements were categorized as wide (> 2 mm) or narrow (≤ 2 mm) [18]. For assessment of the VMT, a #30 K-file (Dentsply Maillefer) was penetrated in the tissue after local anesthesia until touching the bone crest, and the thickness was measured with a ruler to the nearest millimeter [19]. Measurements were also categorized as a thin (≤ 2 mm) or thick (> 2 mm) mucosa.

The surgery duration was recorded in minutes, ranging from the local infiltrative anesthesia until tissue suture. The need for osteotomy for alveolar bone reduction was also recorded.

### Data analysis

Descriptive statistics and between-group comparisons were performed to test differences in baseline characteristics of the two groups. Then, we tested the effect of the different follow-up times on the patient’s perceived outcomes after surgery in the two overdenture groups. The Shapiro-Wilk test was used for testing the normality of data. Non-normal distribution measurement data were expressed as median (interquartile range) and analyzed by non-parametric comparison tests—Mann-Whitney test for between-group comparisons, and the Wilcoxon signed rank test for within-group comparisons. The effect size for longitudinal comparisons was calculated (ES = *Z*/√*N*).

Since each subject gave multiple responses in a repeated-measures design, the assumption of independence of ratings was violated, due to an idiosyncratic factor that affects all responses from the same subject, thus rendering these different responses inter-dependent (correlated) rather than independent. Therefore, a generalized estimating equations (GEE) regression was performed for each of the postsurgical outcomes, considering the combined subject and time-point variables as the within-subject variable to identify the repeated measures across the dataset. The gamma with Log-link was selected as the distribution-link function combination for the dependent variables due to the skewed distribution of data. Due to the excessive occurrence of zero values in the outcome variable (zero-inflated data), added a positive constant (*c* = 10) to all observations “*Y*”, so that *Y* + *c* > 0, preventing the exclusion of the corresponding case in the analysis. GEE models were also tested considering the remission of symptoms (score = 0) for outcome measure, using binary logistic regression. The follow-up period, overdenture group, and clinical features were used as fixed predictor variables in the GEE models. The maximum likelihood estimates were used to calculate the regression parameters, and the Wald statistics were used to test the significance of the model effects. The IBM-SPSS was used for data analysis, and the 0.05 level of significance was considered for statistical inferences.

## Results

**Fig. 1:**
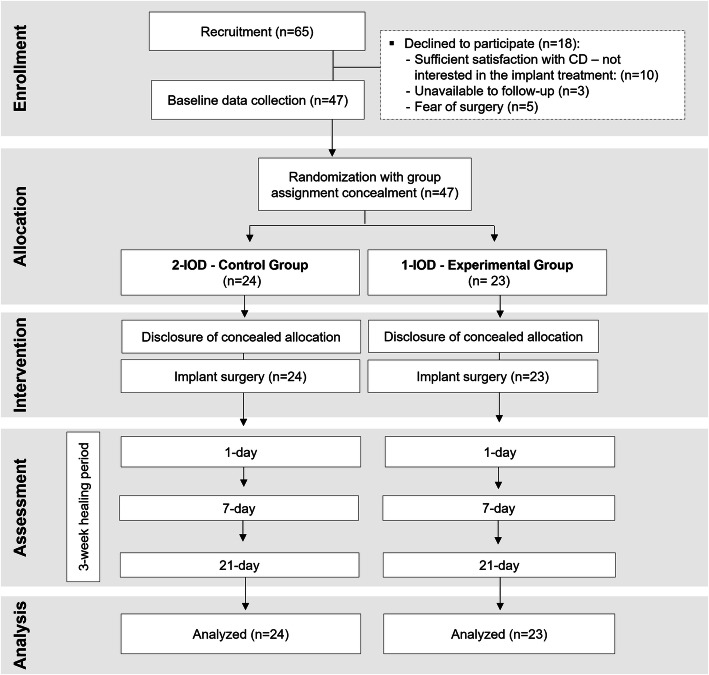
Flowchart of the study

The complete participant flowchart is detailed in Fig. 1. This study included 47 participants who were randomized to the study groups: 1-IOD = 23, and 2-IOD = 24 subjects. Table 1 shows that the two groups were similar regarding age, gender, and clinical features. Differences were found only for the width of keratinized tissue in the lingual side (lower for the 1-IOD group; *p* = 0.040) and for the lingual mucosa thickness (lower for the 2-IOD group; *p* = 0.026).

**Table 1:** Frequency distributions and comparisons of baseline patient characteristics and local clinical features of the study groups

	Overall (*n* = 47)	1-IOD (*n* = 23)	2-IOD (*n* = 24)	*p*-value
Age in years^a^	65.4 (8.6)	66.9 (7.0)	64.0 (9.8)	0.253
Gender (female)	35 (74.5)	15 (65.2)	20 (83.3%)	0.154
Diabetes	18 (38.3)	9 (39.1)	9 (37.5)	0.908
Smokers and former smokers	28 (59.6)	13 (56.5)	15 (62.5)	0.676
Classification of residual ridge forms				
Well-rounded	3 (6.4)	2 (8.7)	1 (4.2)	0.175
Knife-edge	19 (40.4)	6 (26.1)	13 (54.2)	
Flat ridge	8 (17.0)	6 (26.1)	2 (8.3)	
Depressed	17 (36.2)	9 (39.1)	8 (33.3)	
Keratinized tissue width (KTW) ^a^				
Buccal	2.02 (1.44)	1.76 (1.18)	2.27 (1.65)	0.230
Lingual	1.88 (1.33)	1.48 (1.03)	2.27 (1.49)	0.040
Crestal	1.85 (0.91)	1.72 (0.89)	1.98 (0.94)	0.332
Vertical mucosa thickness (VMT) ^a^				
Buccal	2.20 (0.91)	2.35 (0.93)	2.05 (0.88)	0.271
Lingual	1.97 (1.05)	2.30 (1.21)	1.61 (0.72)	0.026
Crestal	2.32 (0.86)	2.33 (0.94)	2.31 (0.79)	0.941
Height of the anterior mandible (mm)^a^	27.7 (3.6)	28.3 (3.5)	27.2 (3.6)	0.278
PDI classification				
II	2 (4.3)	2 (8.7)	0 (0)	0.275
III	40 (85.1)	18 (78.3)	22 (91.7)	
IV	5 (10.6)	3 (13.0)	2 (8.3)	

^a^Mean (and standard deviation)

During the follow-up period after implant placement, three participants of the 2-IOD group had early implant failure, one implant failure for each participant, and a new implant was inserted after 3 months. For data analysis, only the first implant surgery and 3-week healing period were considered for analysis. Implant surgery included osteotomy for alveolar bone reduction in 19 cases (82.6%) of the 1-IOD group and 21 cases (87.5%) of the 2-IOD group (*p* = 0.638). The duration of the surgery was longer for the 2-IOD group (1:34 h ± 0:11) compared to the 1-IOD group (1:09 h ± 0:17), representing a mean 26.6% longer time for insertion of the two implants (*p* < 0.001).

**Fig. 2:**
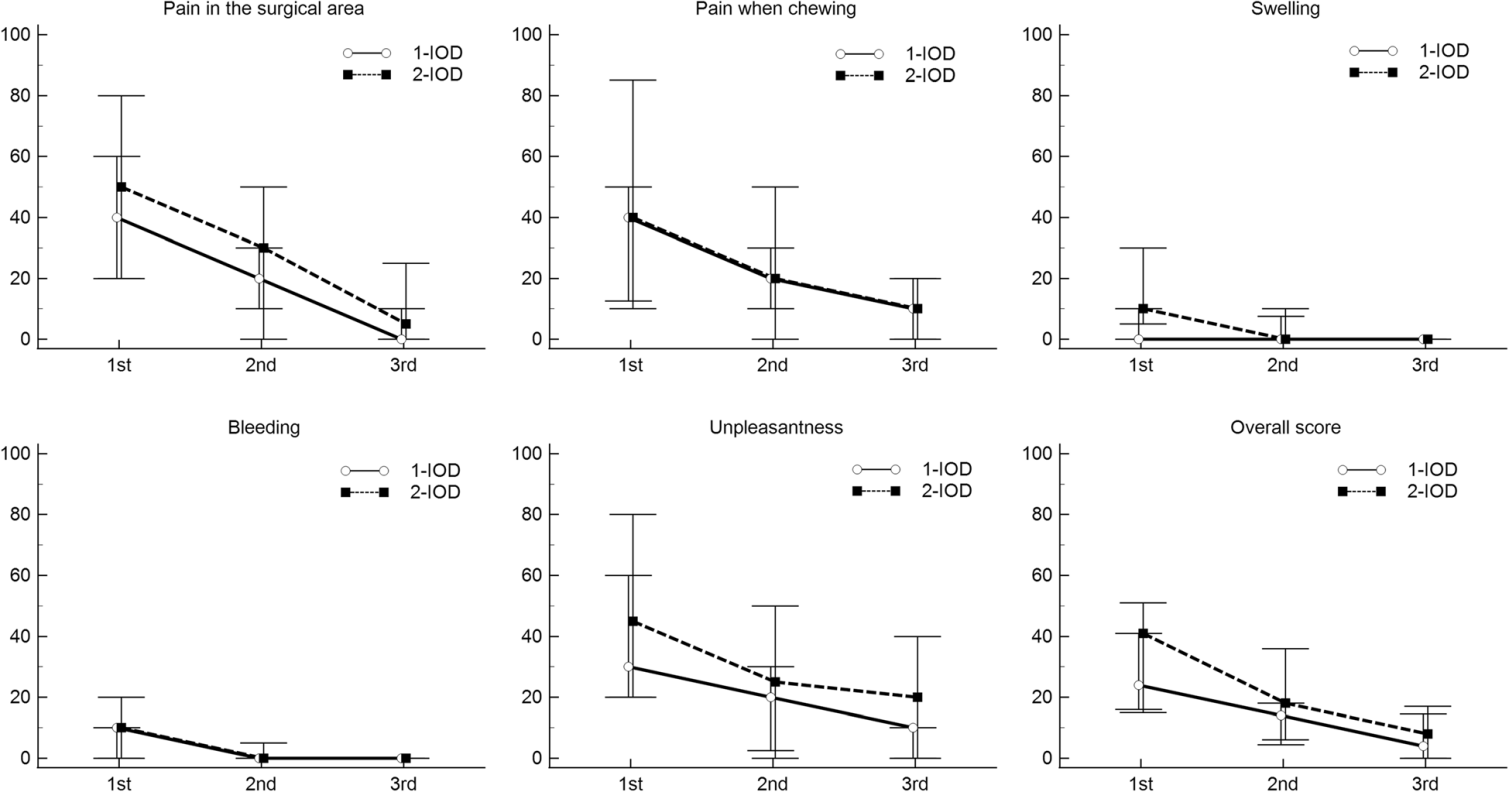
Changes in postsurgical outcomes according to the follow-up period and treatment group

Figure 2 depicts the changes in each of the selected outcomes according to the postsurgical visits and treatment groups. Patient perception of the postsurgical outcomes showed marked progressive improvement for the two groups according to the follow-up periods. Moreover, Table 2 reveals no between-group differences for all outcomes in the assessed time-points, except for a marginally significant higher reporting of swelling in the 2-IOD group in the first 24 h after implant placement (*p* = 0.045). No difference between the 1- and 2-IOD groups was found for the overall patient scores that encompass the mean value of all perceived surgical outcomes.

**Table 2:** Patient perception of postsurgical outcomes according to the follow-up periods and treatment groups. Data are expressed as median (interquartile) values

Outcomes	Groups	Follow-ups			24 h–7 days		7–21 days	
		24-h	7-day	21-day	*p*-value^b^	Effect size^b^	*p*-value^b^	Effect size^b^
Pain in the surgical area	1-IOD	40 (40)	20 (20)	0 (10)	0.012	− 0.53	< 0.001	− 0.74
	2-IOD	50 (65)	30 (50)	5 (28)	0.001	− 0.69	0.002	− 0.64
	*p*-value^a^	0.243	0.400	0.188				
Pain when chewing	1-IOD	40 (40)	20 (20)	10 (20)	0.006	− 0.57	0.059	− 0.39
	2-IOD	40 (78)	20 (50)	10 (20)	0.001	− 0.66	0.008	− 0.54
	*p*-value^a^	0.472	0.820	0.812				
Swelling	1-IOD	0 (10)	0 (10)	0 (0)	0.020	− 0.49	0.439	− 0.16
	2-IOD	10 (28)	0 (10)	0 (0)	0.011	− 0.52	0.067	− 0.37
	*p*-value^a^	0.045	0.361	0.706				
Bleeding	1-IOD	10 (10)	0 (0)	0 (0)	0.003	− 0.62	0.713	− 0.08
	2-IOD	10 (20)	0 (8)	10 (10)	0.009	− 0.54	0.020	− 0.48
	*p*-value^a^	0.669	0.320	0.307				
Unpleasantness	1-IOD	30 (40)	20 (30)	10 (10)	0.008	− 0.55	0.098	− 0.35
	2-IOD	45 (60)	25 (50)	20 (40)	< 0.001	− 0.73	0.033	− 0.44
	*p*-value^a^	0.243	0.536	0.474				
Overall score	1-IOD	24 (26)	14 (14)	4 (16)	< 0.001	− 0.76	0.009	− 0.54
	2-IOD	41 (37)	18 (32)	8 (17.5)	< 0.001	− 0.82	0.001	− 0.68
	*p*-value^a^	0.365	0.353	0.443				

^a^Mann-Whitney test

^b^Wilcoxon signed ranks test

Conversely, Table 2 also shows a significant decrease in scores were observed for the two groups between the 24-h and 7-day follow-ups, and between the 7- and 21-day follow-ups, except for swelling (1- and 2-IOD groups) and bleeding (1-IOD group). For the significant within-group paired comparisons, effect sizes ranged from moderate (< 0.3) to large (< 0.50). The small effect sizes for swelling and bleeding in the 1-IOD group suggest that patients reached a level of no symptoms regarding these aspects in the 7-day follow-up. Consequently, no changes occurred prospectively. These findings suggest that symptoms related to swelling and bleeding were remised earlier in the 1-IOD group.

However, further analysis in Table 3 using GEE to account for the non-independence of data showed that groups were similar concerning all symptoms and the overall score (*p* > 0.05). Nevertheless, as expected, progressive improvement of symptoms occurred from the 24-h to the 7-day and 21-day follow-ups (*p* < 0.001). Similar results were found for the progression to the complete remission of symptoms concerning the evidence of no difference between groups and progressive improvement in the 7-day and 21-day follow-ups (Table 4).

**Table 3:** Regression estimates for the symptoms outcomes according to the treatment groups and follow-up periods. Data are regression coefficients (and *p*-values)

Outcomes	Groups	Outcomes					
		Pain in the surgical area	Pain when chewing	Swelling	Bleeding	Unpleasantness	Overall score
Treatm. groups	1-IOD	0	0	0	0	0	0
	2-IOD	0.235 (0.157)	0.107 (0.543)	0.023 (0.902)	− 0.171 (0.340)	0.166 (0.321)	0.108 (0.443)
Follow-ups	24-h	0	0	0	0	0	0
	7-day	− 0.358 (< 0.001)	− 0.330 (< 0.001)	− 0.559 (< 0.001)	− 0.693 (< 0.001)	− 0.366 (< 0.001)	− 0.415 (< 0.001)
	21-day	− 1.008 (< 0.001)	− 0.692 (< 0.001)	− 0.794 (< 0.001)	− 0.748 (< 0.001)	− 0.663 (< 0.001)	− 0.777 (< 0.001)

**Table 4:** Frequency distribution of the remission of symptoms (symptom score = 0), and tests of regression models for the treatment groups and follow-up as predictor variables

Follow-up	Pain in the surgical area		Pain when chewing		Swelling		Bleeding		Unpleasantness	
	1-IOD	2-IOD	1-IOD	2-IOD	1-IOD	2-IOD	1-IOD	2-IOD	1-IOD	2-IOD
1-day	3 (13.0)	2 (8.3)	4 (17.4)	5 (20.8)	13 (56.5)	6 (25.0)	11 (47.8)	9 (37.5)	3 (13.0)	3 (12.5)
7-day	5 (21.7)	7 (29.2)	5 (21.7)	7 (29.2)	17 (73.9)	14 (58.3)	20 (87.0)	18 (75.0)	6 (26.1)	8 (33.3)
21-day	15 (65.2)	12 (50.0)	11 (47.8)	11 (45.8)	21 (91.3)	21 (87.5)	22 (95.7)	24 (100)	10 (43.5)	11 (45.8)
*p*-value^a^	0.616		0.780		0.056		0.427		0.762	

^a^Tests of model effects for the difference between groups (1-IOD vs 2-IOD). All *p*-values for the effects of the follow-up assessments were < 0.001

Therefore, findings suggest that the progressive resolution of symptoms occurred similarly in the two groups. None of the independent variables listed in Table 1 was significantly associated with the longitudinal changes in symptoms when included as predictor variables in the tested GEE regression models.

## Discussion

This study compared the incidence and severity of postoperative symptoms after implant surgery for treatment with mandibular overdenture retained by one or two implants. Overall reported symptoms were mild and self-limited, resulting in high rates of complete remission after the early loading period of 3 weeks. Moreover, the hypothesis that the more conservative approach using a single implant and the consequent less extensive surgical access and manipulation and shorter surgery time would result in fewer postsurgical symptoms was not confirmed. Findings from this study corroborate previous studies showing that, similarly to the 2-IOD, the 1-IOD presents low morbidity and few postoperative complications [8, 15].

There is scarce information on the comparative short-term outcomes in overdenture treatments, especially related to the number of implants. A previous randomized clinical trial by Mundt et al. [15] compared different loading protocols (immediate versus delayed) for single-implant overdentures and concluded that immediate loading evoked higher postoperative symptoms from the first day and more swelling from the third day after implant surgery than the delayed loading (3-month healing period). Ribeiro et al. [20] investigated the 1-week postoperative period after inserting 2 or 4 mini-implants or 2 conventional diameter implants. They found that the use of 4 implants was associated with increased perception of pain, irrespective of the use of osteotomy and flapped surgical approach, suggesting that postsurgical pain may be more related to tissue manipulation and damage than the implant size/diameter or the number of inserted implants [20]. We also found that the number of implants and the frequency of need for osteotomy for the alveolar bone reduction were similar in the two groups and were not associated with the levels of postoperative symptoms. Moreover, the severity of symptoms may be related to the technical quality of the procedures and the proper management of the bone and soft tissues, even if more extensive procedures are performed.

Pain levels after implant insertion usually achieve maximum intensity reported as mild levels from 6 to 24 h after surgery [6], and some limitations of daily activities and symptoms are expected to occur, particularly during the first 3 postoperative days [21]. A short-term (1-week) prospective study [22] showed that patient’s anxiety and state of anxiety scores affected pain intensity 1 day after implant insertion and were higher for women and associated with a larger number of implants inserted. Patients with higher pain scores after 1 week were those with higher scores at the previous time points. Another study [23] suggested a significant association between swelling and older patients, the placement of more than four dental implants, and surgeries demanding sinus lift or bone regeneration procedures. Furthermore, Klages et al. [24] suggested that during stressful dental procedures, patients with dental anxiety and pain sensitivity above median levels are especially at risk of overstated pain expectations and dental fear, and high fearful patients with a high level of pain sensitivity were found to predict stronger affective, sensory, and intensive pain [24].

Other factors can also influence the postoperative period, related both to the procedure itself and to the patient. Factors inherent to the patient may involve the patient’s self-care in the postoperative period, the correct use of the prescribed medication, and psychological factors, such as anxiety [9, 10, 25]. Postoperative pain is also influenced by the surgical technique and is expected to be less intense with minimal invasive of flapless approaches [12], although there is no sound evidence on the effects of the flapless technique on the occurrence of postoperative infection or marginal bone loss around the implant [26].

We also investigated the effect of mucosal tissue features on the postsurgical symptoms, considering that the gingival biotype is an essential factor in the formation and maintenance of peri-implant gingival architecture in the healing period [27]. The differences in the vertical thickness of the mucosa and the width of the keratinized tissue between 1-IOD and 2-IOD groups do not seem to influence the level of postoperative discomfort. Nevertheless, the long-term maintenance of the quality and thickness of the gingival tissues may be essential for the preservation of the crestal bone level with minimal remodeling and prevention of the incidence of periimplantitis [19].

This study was based on patient reporting of symptoms, which can have multidimensional aspects influenced by several physiological and psychological features. Hence, the role of other factors such as cognitive, psychological, and emotional aspects may be considered to affect the mechanisms behind postoperative pain following implant surgery, and further studies evaluating their isolated and interplayed influence may be warranted. Other clinical factors assessed in this study were considered to be fairly distributed between groups during the randomization process, such as gender, smoking habits, age, and osteotomy for alveolar bone reduction, and were not found to be determinants of the reporting of symptoms. In addition, although the evolution of symptoms occurs in a continuum, similarly to other studies, we planned the follow-up time points according to the pre-scheduled visits for dressing (24 h), suture removal (7 days), and implant loading (21 days) and not demanding additional visits for the patient for data collection.

Regarding implant surgery outcomes, three implant failures occurred in the 2-IOD group, which were attributed to low primary stability and excessive trauma due to the denture’s use during the healing period. However, the failures were detected only during the removal of the healing caps for insertion of the overdenture abutment 3 weeks after implant insertion. They were not associated with higher pain levels by patient reporting. The higher incidence of implant failure in the 2-IOD group could be related to the lateral position of the implants, more prone to higher functional distress than the midline region.

Finally, this study provides additional information on the comparative effectiveness of the 1-IOD compared to the 2-IOD. Although previous studies have shown positive aspects and similar outcomes using both treatment strategies, clinical decisions regarding selecting one treatment over another may rely on comprehensive factors that include patient preferences and expectations, treatment costs, and specific clinical characteristics. Therefore, the option for a single-implant overdenture may be justified by the patient’s preference for a treatment that is less invasive, less costly, and easy to maintain over time. The current body of evidence on this topic may guide the decision-making process for the individual patient and subside clinical practice in various settings, and further research.

## Conclusion

Within the limits of this short-term clinical trial, it was concluded that, although the 1-IOD may be potentially associated with lower surgical time, more conservative bone drilling, and flap extension, it seems not to be significantly advantageous from the patient perspective regarding the level of postsurgical symptoms and may not be a determinant factor in treatment decision-making.

## Data Availability

Data are available from the authors upon reasonable request.
